# The National Magazine for January

**Published:** 1890-03

**Authors:** 


THE NATIONAL MAGAZINE for January announces two new and valuable departments—"Biblical Literature," and "Pedagogy"—with Rev. J. C. Quinn, Ph. D., and J. S. Mills, A. M., President of Western College, as editors.

Agricultural readers will be especially interested in the new "Institute of Agriculture," described in this number—a part of the University Extension System of the National University of Chicago, whose non-resident or correspondence undergraduate and post-graduate courses have met with such favor. Other articles are by Professor E. A. Birge, of the University of Wisconsin, and eminent specialists. Published at 147 Throop Street, Chicago, Ill. Subscription, $1.00 per year. Sample copy, 10 cents. Three cash prizes of fifty dollars each for the best essays on "Our Common Schools," "Study of the Bible," "How to Keep Young Men on the Farm," are announced.

